# Mobile Insight in Risk, Resilience, and Online Referral (MIRROR): Psychometric Evaluation of an Online Self-Help Test

**DOI:** 10.2196/19716

**Published:** 2020-09-25

**Authors:** Merel Marjolein van Herpen, Manon A Boeschoten, Hans te Brake, Niels van der Aa, Miranda Olff

**Affiliations:** 1 ARQ Centre of Expertise for the Impact of Disasters and Crises Diemen Netherlands; 2 Department of Psychiatry Amsterdam Neuroscience & Public Health Amsterdam University Medical Center Amsterdam Netherlands; 3 ARQ Centre ‘45 Oegstgeest/Diemen Netherlands; 4 ARQ National Psychotrauma Centre Diemen Netherlands

**Keywords:** potentially traumatic events, mobile mental health, self-help, online, resilience, posttraumatic stress disorder

## Abstract

**Background:**

Most people who experience a potentially traumatic event (PTE) recover on their own. A small group of individuals develops psychological complaints, but this is often not detected in time or guidance to care is suboptimal. To identify these individuals and encourage them to seek help, a web-based self-help test called Mobile Insight in Risk, Resilience, and Online Referral (MIRROR) was developed. MIRROR takes an innovative approach since it integrates both negative and positive outcomes of PTEs and time since the event and provides direct feedback to the user.

**Objective:**

The goal of this study was to assess MIRROR’s use, examine its psychometric properties (factor structure, internal consistency, and convergent and divergent validity), and evaluate how well it classifies respondents into different outcome categories compared with reference measures.

**Methods:**

MIRROR was embedded in the website of Victim Support Netherlands so visitors could use it. We compared MIRROR’s outcomes to reference measures of PTSD symptoms (PTSD Checklist for DSM-5), depression, anxiety, stress (Depression Anxiety Stress Scale–21), psychological resilience (Resilience Evaluation Scale), and positive mental health (Mental Health Continuum Short Form).

**Results:**

In 6 months, 1112 respondents completed MIRROR, of whom 663 also completed the reference measures. Results showed good internal consistency (interitem correlations range .24 to .55, corrected item-total correlations range .30 to .54, and Cronbach alpha coefficient range .62 to .68), and convergent and divergent validity (Pearson correlations range –.259 to .665). Exploratory and confirmatory factor analyses (EFA+CFA) yielded a 2-factor model with good model fit (CFA model fit indices: χ^2^_19_=107.8, *P*<.001, CFI=.965, TLI=.948, RMSEA=.065), conceptual meaning, and parsimony. MIRROR correctly classified respondents into different outcome categories compared with the reference measures.

**Conclusions:**

MIRROR is a valid and reliable self-help test to identify negative (PTSD complaints) and positive outcomes (psychosocial functioning and resilience) of PTEs. MIRROR is an easily accessible online tool that can help people who have experienced a PTE to timely identify psychological complaints and find appropriate support, a tool that might be highly needed in times like the coronavirus pandemic.

## Introduction

Most people will experience at least one potentially traumatic event (PTE) in their lives [1-5]. The impact of PTEs is not the same for every individual. Research shows that most individuals are able to maintain a healthy level of functioning or resilience after experiencing a PTE and psychological complaints usually diminish over time without professional support [1,6-10]. However, a small but significant group of individuals develops psychological complaints such as posttraumatic stress disorder (PTSD) that require care [2].

Experiencing psychological complaints a few days to weeks after a PTE is often considered normal [11-13]. The National Institute for Health and Care Excellence (NICE) advises to consider active monitoring—also known as watchful waiting—following a PTE (ie, regular monitoring of people with some PTSD symptoms within 1 month of the event) [14]. The European Network for Traumatic Stress (TENTS) guideline for post-disaster psychosocial care advises against formal screening of everyone affected by a PTE but stresses the importance of identifying individuals in need of support. Once PTSD has been diagnosed, early treatment is advised [14-18]. It could be concluded, then, that support for people who have experienced a PTE is necessary, preferably early, and easily accessible.

Unfortunately, the small but significant group that develops persisting psychological complaints is often not detected in time or guidance to care is suboptimal [19,20]. Guidance to care can be hindered due to people not recognizing their symptoms or having self-stigma, which prevents them from seeking help [21-24]. In addition, health care facilities may lack the resources to be able to reach people who have experienced a PTE and identify the ones who need support [23,25]. Also, general practitioners may not recognize PTSD symptoms [26] or other psychological complaints [27].

In order to prevent the development and persistence of trauma-related complaints, timely and accurate identification is needed [23,28]. Short and easy-to-use screening instruments could enable individuals at risk of developing psychological complaints to self-identify and monitor possible symptoms after PTEs. Moreover, providing online or mobile self-help tests can aid in timely identification of symptoms in people who have experienced a PTE, providing more information regarding normal psychological responses and encouraging help seeking [29,30].

Multiple studies show that when one chooses to assist people who have experienced a PTE, it is important to support self-reliance and resilience [1,11,14]. Normalizing and validating emotional responses can promote the capacity to deal with these emotions [11]. Also, the extent to which individuals identify themselves as being resilient is considered to positively influence post-trauma outcomes [31,32]. Several self-report screening instruments are available to predict PTSD, such as the Trauma Screening Questionnaire, Impact of Event Scale–Revised or PTSD Checklist for the *Diagnostic and Statistical Manual of Mental Disorders, Fifth Edition* (PCL-5) [33,34]. However, most instruments only screen for complaints and do not inquire about protective factors such as psychological resilience and psychosocial functioning [33,34]. In addition, most screening instruments do not consider the time period that has passed since the event. Such information is necessary to determine whether reported complaints can be appraised as normal given the stressful event just happened or whether referral to care is needed [14]. By not including time in classifying responses, screening can overlook or misappraise the different response trajectories that have been found after PTEs [9].

To incorporate above guideline advice and address the aforementioned concerns in the early support of people who have experienced a PTE, Mobile Insight in Risk, Resilience, and Online Referral (MIRROR) was developed. MIRROR is a web-based self-help test with the potential to reach large groups of people who are seeking reassurance on how they are coping. MIRROR takes an innovative approach since it integrates both negative and positive outcomes of PTEs and time since the event. This was realized by creating a new questionnaire based on existing measures on resilience, functioning, and PTSD, and by developing a new algorithm that takes into account multiple factors. In compliance with NICE, TENTS, and DSM-5 guidelines [14,15,35], MIRROR’s algorithm includes the following as main weight factors: severity of complaints, time passed since event, and level of psychosocial functioning. MIRROR provides users with personal advice based on respondent answers with relevant follow-up support options such as a reminder for self-monitoring and contact information for consultation. Giving personal feedback to users is recommended to augment the use of mobile self-tests after PTEs [36]. Also, arranging active monitoring with follow-up within 1 month is advised [14]. Of relevance, no difference has been found between responses on a PTSD self-report administered via a mobile device versus paper administration [37]. MIRROR aims to contribute to the early identification of those likely to develop psychological complaints and encourage them to seek help. At the same time, MIRROR aims to support self-reliance by facilitating self-monitoring and self-recovery through follow-up support options.

While it is recognized that mobile apps have the potential to improve timely identification of complaints and delivery of mental health support after PTEs, there is very little research on their validity, reliability, and effectiveness [29,30,38,39]. Therefore, the aims of this study were to assess MIRROR’s use, examine MIRROR’s psychometric properties (factor structure, internal consistency, and convergent and divergent validity) and evaluate how well MIRROR classifies respondents into different outcome categories compared with reference measures.

## Methods

### Mobile Insight in Risk, Resilience, and Online Referral (MIRROR)

A multidisciplinary team of professionals in the fields of psychotrauma (clinicians, researchers, and policy officers) and victim and crisis support developed MIRROR. The items and algorithm were based on existing protocols—DSM-5 and the *International Statistical Classification of Diseases and Related Health Problems, Tenth Revision* (ICD-10) [35,40]—best practices and recommendations of the Dutch National Multidisciplinary Guideline on Psychosocial Support in Disasters and Crises [41], and international guidelines for PTSD and postdisaster psychosocial care [14,15].

MIRROR consists of 2 parts. Part 1 includes items regarding event-related characteristics: type of event, measured with all events of the Dutch version of the Life Events Checklist for the DSM-5 (LEC-5) [42], time passed since the event (measured in weeks), and relation to the event (happened to me, learned about it, witnessed it, part of my job). Part 2 consists of 8 items divided in 3 sections. The first concerns PTSD core symptoms (4 items in total; 1 about intrusion, 2 about avoidance, and 1 about arousal). The items are developed based on the clusters in the DSM-IV, DSM-5, ICD-10, and ICD-11. Higher scores reflect more PTSD symptoms. The second concerns the item “how would you rate your present functioning (at work/home),” based on the widely used Global Assessment of Functioning (GAF) score for which higher scores reflect a higher level of functioning. The third concerns resilience (3 items in total; about social support, self-reliance, and problem solving), based on the resilience concept as introduced by Van der Meer et al [43]. Higher scores reflect more resilience. PTSD and resilience items are answered on a 5-point response scale, ranging from 1 (never) to 5 (all the time). Functioning is rated on a scale from 1 to 10.

MIRROR’s algorithm aims to identify PTSD symptoms, psychosocial functioning, and resilience; normalize complaints (ie, reassuring users that it is normal to experience distress shortly after a PTE); and stimulate seeking support in users with persisting complaints. See Multimedia Appendix 1 for an overview of the possible outcomes of the algorithm. In the algorithm, MIRROR’s PTSD scale and functioning item are classified in 3 levels: low, moderate, and high. Resilience is categorized as either low or high. The categorizations are based on the aforementioned existing protocols and best practices. MIRROR’s algorithm differentiates 3 phases of time passed since the event: (1) less than 1 week ago, (2) between 1 and 4 weeks, and (3) more than 4 weeks or reoccurring. These were based on the assumption that complaints after PTEs may occur but will generally diminish over time, as most people recover on their own [6]. Therefore, the occurrence of PTSD core complaints with moderate to low functioning shortly after an adverse event can be seen as normal [11-13], but if complaints and moderate to low functioning are present after 1 month, guidance to care is needed [14-18].

MIRROR summarizes the outcome of its algorithm to respondents as either green, orange, or red. Together with this color outcome, respondents receive personal advice. The color outcome is based on the level of complaints, functioning, and time passed since the event. MIRROR’s resilience scale is not included in the color outcome because based on current research it is unclear precisely how resilience interacts with the development of PTSD complaints and functioning after PTEs. Nonetheless, resilience is integrated in the personal advice to stimulate the use of social support. If respondents score low on resilience they are encouraged to seek support from those close to them and individuals who have experienced similar events.

A green outcome indicates few complaints and/or sufficient functioning, and the accompanying advice states no further action is needed. An orange outcome indicates complaints and moderate functioning in combination with a PTE that happened only recently (ie, less than 1 month). The accompanying advice is directed at normalizing complaints combined with promoting watchful waiting and encouraging setting a reminder to use MIRROR again in 2 weeks to assess if complaints have diminished. The red outcome indicates significant complaints (ie, low functioning or complaints with moderate to low functioning for a longer period or due to a reoccurring event) which have persisted for more than 1 month. Therefore, the advice aims to encourage the user to seek consultation with a general practitioner or to contact Victim Support Netherlands. MIRROR provides follow-up support options with its advice, such as the opportunity to get in touch with people who have had similar experiences, reading information about dealing with stress reactions, or setting a reminder to use MIRROR again in 2 weeks.

### Participants and Procedure

MIRROR was available in the Dutch language and open for each visitor on the website of Victim Support Netherlands (Slachtofferhulp Nederland). The specifically targeted sample consisted of website visitors who were automatically led to MIRROR when searching for information regarding stress reactions following a PTE. MIRROR is a responsive website; respondents did not have to download it. MIRROR can be used on mobile and nonmobile devices. To evaluate the psychometric properties of MIRROR, we added a research survey with reference measures (see details in Measures) after the MIRROR questions. Data collection took place during a period of 6 months. We tested the usability and technical functionality of MIRROR and the research survey before making it available. Each item was presented on a new webpage.

Before starting MIRROR, respondents were invited to participate in the research survey. Participants were informed regarding the purpose of the study, duration time of the survey, and data storage. Participation was voluntary and completely anonymous. Respondents received no incentive for completing MIRROR or the research survey. They were asked for informed consent to use their data for research purposes, in accordance with the European General Data Protection Regulation. The Medical Ethical Committee of Amsterdam University Medical Center exempted this study from formal review (W18_364 #18.435).

Data collection took place between February and August 2019. Only original answers were saved in the database. That is, if respondents went back to change their answers once they already received their advice, changes were not saved. We followed data cleaning recommendations by Birnbaum [44] and Wood et al [45]. Data were discarded when respondents did not complete all survey items. In case of identical answers on all items of the different reference measures, other systematic answering patterns, or obvious unusual missing answers on certain measures, we reviewed individual results thoroughly and discarded the data in case of doubt.

### Measures

#### Posttraumatic Stress Disorder Symptoms

To measure PTSD symptoms, we used the Dutch version of the PCL-5 [46,47]. The PCL-5 consists of 20 items and measures symptoms of intrusion (cluster B, 5 items), avoidance (cluster C, 2 items), negative alterations in cognitions and mood (cluster D, 7 items), and alterations in arousal and reactivity (cluster E, 6 items) in the past month. All items are answered on a 5-point scale, ranging from 0 (not at all) to 4 (extremely). The PCL-5 showed good psychometric properties in different languages [48-50]. The total score was calculated by adding all item scores. Scale scores per cluster were calculated by adding the scores of the corresponding items. Higher scores reflect more severe symptoms. Cronbach alphas in our sample ranged between .77 and .86 for the B, C, D, and E clusters. The DSM-5 rule to determine a provisional PTSD diagnosis was followed. This entails treating each item with a minimum score of 2 as a symptom endorsed and requiring at least one B symptom, one C symptom, two D symptoms, and two E symptoms [46].

#### Depression, Anxiety, and Stress

To assess other common psychological complaints after PTEs, we used the Dutch short version of the Depression Anxiety Stress Scale (DASS-21) measuring depression (7 items), anxiety (7 items), and stress (7 items) [51,52]. The DASS-21 is a valid and reliable measure [53,54]. Item scores were summed to calculate scale scores and the total score. Higher scores reflect more severe symptoms. In our sample, Cronbach alphas were .92, .86, and .86 for depression, anxiety, and stress scales, respectively. A 4-point response scale measures the extent to which each state has been experienced over the past week ranging from 0 (did not apply to me at all) to 3 (applied to me very much, or most of the time). To determine cutoff values, DASS-21 scale scores were multiplied by two, in accordance with the scale’s manual [52]. The manual provides cutoff scores for a Dutch clinical sample. These discriminate the following categories: normal (depression <9, anxiety <7, stress <14), mild (depression 10-13, anxiety 8-9, stress 15-18), moderate (depression 14-20, anxiety 10-14, stress 19-25), severe (depression 21-27, anxiety 15-19, stress 26-33) and extremely severe (depression >28, anxiety >20, stress >34).

#### Psychological Resilience

We used the Resilience Evaluation Scale (RES) to assess psychological resilience [43]. The 9 items are rated on a 5-point scale ranging from 0 (strongly disagree) to 4 (strongly agree). We calculated the total score by adding all items. Higher scores reflect more psychological resilience. The RES is a valid and reliable measure [43]. In this sample, Cronbach alpha of the total scale was .88.

#### Positive Mental Health

We assessed positive mental health with the Dutch version of the Mental Health Continuum Short Form (MHC-SF) [55,56]. The MHC-SF measures emotional well-being (3 items), social well-being (5 items), and psychological well-being (6 items). Items were rated on a 6-point scale ranging from 0 (never) to 5 (every day). The MHC-SF is a valid and reliable instrument [56,57]. We calculated the total score by summing all item scores. Higher scores reflect more positive mental health. In this sample, Cronbach alpha of the total scale was .93.

#### Google Analytics

Google Analytics data were collected between March and August 2019 to examine MIRROR’s use. Due to technical problems, data from February 2019 were missing. The data provide information on the number of unique visits per page, type of device used, and number of visitors who have started MIRROR (defined as a unique page visit on MIRROR’s start page) and who have finished MIRROR (defined as unique page visit on MIRROR’s outcome and advice page). Google Analytics cannot determine to what extent the follow-up options were used, but it can detect how many respondents have visited the follow-up support option pages.

### Statistical Analyses

#### Sample and Use

Since participation in the research survey was optional, this resulted in two 2 samples. The MIRROR-only sample consists of respondents who only completed MIRROR. The validation sample includes respondents who completed MIRROR and the accompanying survey with reference measures before receiving their advice. The total sample combines these two samples, consisting of all respondents. To examine if the validation sample was representative of the MIRROR user, we used independent-samples *t* tests in SPSS Statistics version 23 (IBM Corporation) to compare the MIRROR-only sample with the validation sample based on their MIRROR scores and event-related characteristics.

We used the total sample to evaluate MIRROR’s use and examine MIRROR’s factor structure and internal consistency because for these analyses only data from MIRROR were needed. We used the validation sample to examine MIRROR’s convergent and divergent validity and evaluate how well MIRROR classifies respondents into different outcome categories because for these analyses data from MIRROR as well as reference measures from the accompanied survey were needed.

#### Factor Structure

We used Mplus version 8 (Muthen & Muthen) to conduct exploratory factor analysis (EFA) using geomin rotation and confirmatory analysis (CFA). EFA assumes that any item may be associated with any factor. CFA specifies expected relationships between items and their underlying latent factors. Because items of MIRROR’s PTSD and resilience section were categorical, they were treated as ordinal and therefore the means and variance adjusted weighted least square (WLSMV) estimator was used. An underlying normal distribution was assumed for each ordinal item, where the 5 response categories were divided by 4 thresholds estimated from the data. MIRROR’s functioning item has 10 response categories and was treated as continuous. Because MIRROR’s factor structure was not tested before, several models with different numbers of latent factors were examined using EFA. To assess the model with the optimal number of latent factors needed to adequately account for the correlations among item scores, we used Kaiser criterion (ie, eigenvalues of the latent factors >1) and model fit statistics. The model with the best balance between model fit, parsimony, and conceptual interpretability was selected as the most optimal model. Subsequently, CFA was used to test the optimal model based on EFA. The difference in goodness-of-fit between nested models was evaluated with the difftest option in Mplus for appropriate chi-square difference testing with the WLSMV estimator [58]. The chi-square difference test is highly sensitive to sample size such that even trivial differences between two nested models may be significant [59]. Therefore, we also assessed the difference in comparative fit index (CFI). A difference in CFI <0.01 indicates a better fit of the nested model compared with the more complex model [59]. For EFA and CFA, the model fit indices CFI, Tucker-Lewis index (TLI), and root mean square error of approximation (RMSEA) were used to evaluate model fit. Model fit can be considered good when CFI and TLI are close to .95, and RMSEA <.06 [60]. If RMSEA <.08, model fit can be considered adequate [60].

#### Internal Consistency

We evaluated internal consistency of MIRROR’s PTSD and resilience section with interitem correlations, corrected item-total correlations, and Cronbach alpha in SPSS Statistics version 23. Internal consistency of MIRROR’s functioning section could not be evaluated since it is represented by only one item. When most interitem correlations are in the recommended range of .15 to .50 (moderate magnitude) and Cronbach alpha for the scale is >.80, internal consistency can be considered good [61]. Cronbach alpha is a function of scale length and therefore is likely to be lower for MIRROR’s scales since they consist of 3 or 4 items [61]. Corrected item-total correlations were computed to assess whether item scores regarding PTSD and resilience are associated with overall PTSD and resilience scores.

#### Convergent and Divergent Validity

To evaluate MIRROR’s convergent and divergent validity, we calculated Pearson correlations between the MIRROR scales and reference measures. Convergent and divergent validity can be considered good when the correlations between a scale and equivalent measure (eg, MIRROR’s PTSD scale and the PTSD scale of the PCL-5) are significant and high while correlations between this scale and other related measures (eg, MIRROR’s PTSD scale and depression scale of the DASS-21) are lower and moderate or modest in magnitude.

#### Classification Quality

To evaluate how well MIRROR classifies respondents into a red, orange, or green outcome, we tested whether respondents in these three outcome categories differed on related reference measures by using cross-tabs and analysis of variance (ANOVA). If the assumption of equal variances was violated, we used the Welch *F*-test and Games-Howell post hoc test. MIRROR’s PTSD scale score was calculated by summing the 4 PTSD items. Higher scores reflect more severe symptoms. MIRROR’s resilience scale score was calculated by a summing the 3 items. Higher scores reflect more resilience. Provisional PTSD diagnosis based on PCL-5 were used to classify respondents. To examine the distribution on depression, anxiety and stress symptoms, respondents were classified by comparing their scores to a Dutch clinical reference group. Respondents with normal and mild complaints compared with the reference group were classified into one group representing subclinical complaints. Respondents with average, severe, and very severe complaints compared with the reference group were classified into another group, representing clinical complaints. Since no reference groups were available with regard to the RES and MHC-SF, the sample was divided into tertiles (ie, 3 groups of equal size divided by the 33rd and 66th percentile) based on the total scores of the RES and MHC-SF. With regard to the RES, the first tertile (scores ≤17) was assumed to represent relatively low psychological resilience, the second tertile (scores from 18 to 24) relatively moderate psychological resilience, and the third tertile (scores ≥25) relatively high psychological resilience. With regard to the MHC-SF, the first (scores ≤23), second (scores from 24 to 47), and third tertile (scores ≥48) were assumed to represent relatively low, moderate, and high positive mental health, respectively.

## Results

### Sample and Use

MIRROR was completed 1314 times in the study period of 6 months. In total, 51.90% (682/1314) of respondents started the research survey. We deleted 51 respondents who indicated they used MIRROR on behalf of a family member, partner, friend, or colleague who experienced a PTE. We deleted 37 repeated measurements, completed by respondents who set a reminder. We excluded 95 respondents because they did not complete all research survey items. After thorough investigation of the answering patterns, we deleted 19 respondents because of unusual answering patterns. A total of 84.63% (1112/1314) of respondents were retained in the total sample, of whom 59.62% (validation sample, 663/1112) also completed all questionnaires of the accompanying research survey.

Table 1 presents the MIRROR scores, outcomes, and event-related characteristics for the MIRROR-only and validation sample. We found no significant difference between the samples on MIRROR’s PTSD scale: t_1110_=–.401, *P*=.69; resilience scale: t_1110_=.752, *P*=.45; or level of functioning t_1110_=1.547, *P*=.12. We found a significant association between sample and MIRROR outcome: χ^2^_2,n=1112_=18.99, *P*<.001; the validation sample consisted of more respondents with the red MIRROR outcome than the MIRROR-only sample. The event-related characteristics for both samples were similar, see Table 1. Overall, the validation sample can be considered representative of all MIRROR users in this study period. In the validation sample, 74.2% (492/663) of respondents were female. Almost half (300/663, 45.3%) of respondents were aged between 21 and 40 years. Tables 2 and 3 present the frequency distributions for MIRROR’s response categories.

**Table 1:** Mobile Insight in Risk, Resilience and Online Referral (MIRROR) scores, outcomes, and event-related characteristics for the validation sample and MIRROR-only sample.

MIRROR^a^		Validation^b^ (n=663)	MIRROR^b^ only (n=449)
**MIRROR scores, mean (SD)**			
	MIRROR PTSD^c^ scale	14.88 (3.39)	14.80 (3.28)
	MIRROR functioning	4.92 (1.96)	5.11 (1.94)
	MIRROR resilience scale	10.08 (2.36)	10.91 (2.37)
**MIRROR^a^ outcome^b^, n (%)**			
	Red	409 (61.7)	224 (49.9)
	Orange	230 (34.7)	214 (47.7)
	Green	24 (3.6)	11 (2.4)
**Type of event (LEC-5^d^), n (%)**			
	Another very stressful event or experience	216 (32.6)	150 (33.4)
	Transportation accident	115 (17.4)	107 (23.8)
	Physical assault	109 (16.5)	50 (11.1)
	Sudden accidental death	38 (5.7)	20 (4.5)
	Serious accident at work, home, or during recreation	33 (5.0)	28 (6.2)
	Sexual assault	33 (5.0)	18 (4.0)
	Assault with a weapon	30 (4.5)	25 (5.6)
	Other unwanted or uncomfortable sexual experience	30 (4.5)	14 (3.1)
	Sudden violent death	24 (3.6)	16(3.6)
	Severe human suffering	14 (2.1)	5 (1.1)
	Life-threatening illness or injury	10 (1.5)	5 (1.1)
	Fire or explosion	9 (1.4)	4 (0.9)
	Combat or exposure to a war zone	1 (0.2)	0 (0)
	Captivity	0 (0)	4 (0.9)
	Serious injury, harm, or death caused by you to someone else	0 (0)	3 (0.7)
	Natural disaster	0 (0)	0 (0)
**Relation to the event, n (%)**			
	Event happened to me	480 (72.5)	311 (69.3)
	I witnessed the event	129 (19.5)	94 (20.9)
	I learned about the event	42 (6.3)	35 (7.8)
	Other^e^	11 (1.7)	9 (2.0)
**Work-related, n (%)**			
	No	586 (88.4)	379 (84.4)
	Yes	77 (11.6)	70 (15.6)
**Time since the event, n (%)**			
	Less than 1 week	241 (36.3)	218 (48.6)
	Over 4 weeks	214 (32.3)	113 (25.2)
	Between 1 and 4 weeks	144 (21.7)	90 (20.0)
	It happens repeatedly	64 (9.7)	28 (6.2)

^a^MIRROR: Mobile Insight in Risk, Resilience, and Online Referral.

^b^Significant association between sample and MIRROR outcome, *P*<.001.

^c^PTSD: posttraumatic stress disorder.

^d^LEC-5: Life Events Checklist for DSM-5.

^e^If respondents could not select one of the event relations (happened to me, witnessed it, learned about it, work-related), they are asked to specify their relation to the event.

**Table 2:** Frequency distribution in percentages of Mobile Insight in Risk, Resilience and Online Referral (MIRROR) item response categories, items 1-4 and 6-8 (n=1112).

Scale and item number		Never	Rarely	Sometimes	Often	All the time
**PTSD^a^**						
	1	2.7	5.7	16.6	38.5	36.5
	2	5.1	8.5	19.3	27.4	39.6
	3	9.3	13.8	26.9	22.9	27.1
	4	8.5	11.4	26.7	26.8	26.6
**Resilience**						
	6	5.3	8.5	21.7	35.3	29.3
	7	7.3	15.6	35.2	30.5	11.5
	8	5.2	15.1	45.6	28.1	5.9

^a^PTSD: posttraumatic stress disorder.

**Table 3:** Frequency distribution in percentages of Mobile Insight in Risk, Resilience and Online Referral (MIRROR) item response categories, item 5 (n=1112).

Scale and item number		1	2	3	4	5	6	7	8	9	10
Functioning											
	5	4.9	6.9	10.4	15.8	19.8	20.9	12.1	6.3	1.6	1.3

**Figure 1:**
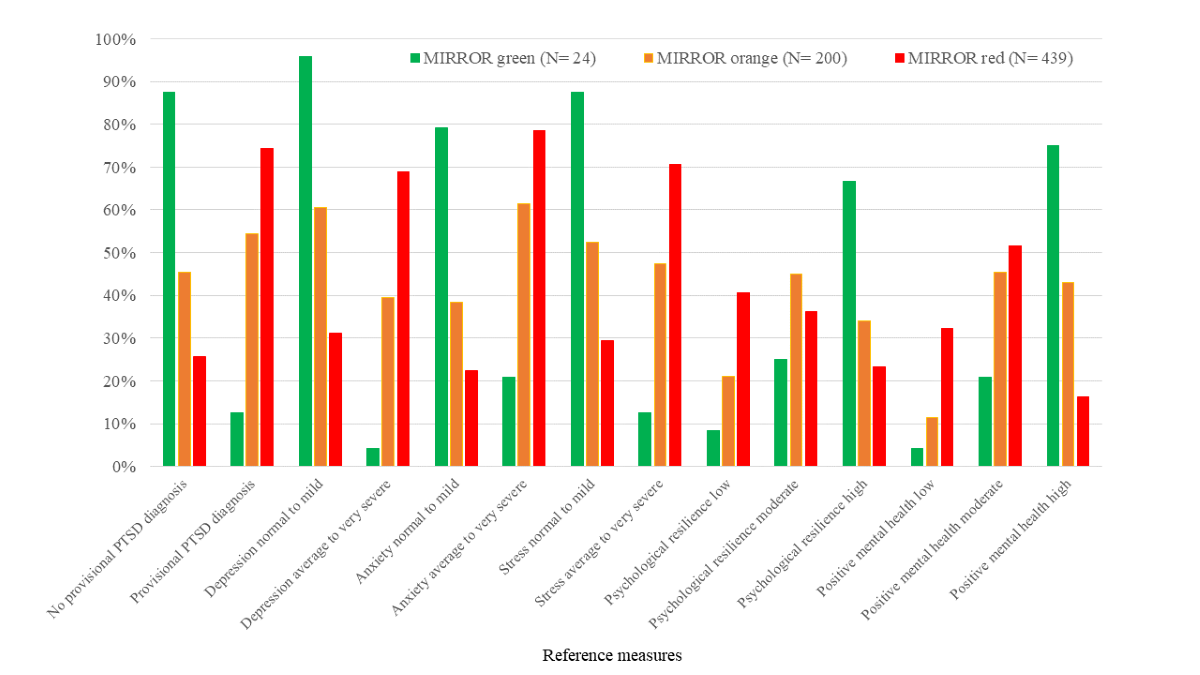
Classification percentages on reference measures of each Mobile Insight in Risk, Resilience, and Online Referral (MIRROR) outcome category.

A detailed overview of the scores of the validation sample on the reference measures can be found in Multimedia Appendix 2. Overall, these show a high level of complaints in our sample and rather low levels of psychological resilience and positive mental health (also see Table 7 and Figure 1 for reference measures of each MIRROR outcome category).

Google Analytics data provided insight into MIRROR’s use. The number of visitors who started MIRROR was 2555, of whom 2247 (87.95%) finished it. The original database contained 1314 entries. This discrepancy can be explained by users having the opportunity to refuse to have their data saved before starting. Of all users, 47.59% (1216/2555) chose this option. Furthermore, of the follow-up support options, the “seek contact with Victim Support Netherlands” page had most views (411 unique views), followed by “more information” (293 unique views), “send your advice to yourself or someone else” (235 unique views), “seek contact with people who have had similar experiences” (209 unique views), and “set a reminder” (161 unique views). A total of 28.7% (113/394) of respondents who received the orange outcome and were advised to complete MIRROR again in 2 weeks immediately set a reminder to complete MIRROR again in 2 weeks. A total of 22.1% (25/113) did so at the time of data analyses. The most often used device was the smartphone (1566/2555, 61.29%), followed by desktop (794/2555, 31.08%), and tablet (195/2555, 7.63%).

### Factor Structure

Table 4 presents the factor loadings for the 2-factor and 3-factor solution model of MIRROR as estimated by EFA. EFA yielded a 3-factor solution with good model fit based on all fit indices. The Kaiser criterion was met for the first 2 factors, eigenvalues of the third through eighth factor were <1. The 3-factor solution separated MIRROR’s PTSD items into 2 factors: 1 with the intrusion item and 1 with the avoidance and arousal/reactivity items. However, item 2 (“have you become jumpy and/or vigilant since the event?”) cross-loaded significantly on 2 factors within the model, with only a small difference between the 2 factor loadings (λ=0.030). This indicates that item 2 did not sufficiently distinguish between both factors. The 3-factor solution clustered the functioning item with the resilience items into a third factor.

**Table 4:** Geomin rotated factor loadings for the 2-factor and 3-factor solution model of Mobile Insight in Risk, Resilience and Online Referral (MIRROR) as estimated by exploratory factor analysis (n=1112).

MIRROR^a^ items	2-factor solution^b^		3-factor solution^c^		
	F1	F2	F1	F2	F3
1. Are you troubled by images of or thoughts about the event?^d^	0.525*	–0.004	0.813*	0.015	0.018
2. Have you become jumpy and/or vigilant since the event?^e^	0.585*	–0.009	0.308*	0.338*	–0.012
3. Do you try to avoid things that are related to the event?^f^	0.789*	0.071	–0.000	1.078*	0.245*
4. Do you try to avoid thinking about the event?^g^	0.648*	–0.016	0.208*	0.459*	–0.019
5. How would you rate your present functioning (at work/home)?^h^	–0.153*	0.354*	–0.213*	0.004	0.360*
6. Do you experience support from those close to you?^i^	0.081*	0.388*	0.160*	–0.064	0.374*
7. Are you confident in yourself?^j^	0.006	0.827*	0.010	–0.021	0.827*
8. Are you able to deal with any problems you encounter?^k^	–0.015	0.730*	–0.074	0.018	0.718*

^a^Mobile Insight in Risk, Resilience and Online Referral.

^b^Model fit indices for the 2-factor solution: χ^2^_13_=88.7, *P*<.001, CFI=.969, TLI=.933, RMSEA=.072.

^c^Model fit indices for the 3-factor solution: χ^2^_7_=12.6, *P*=.084, CFI=.998, TLI=.991, RMSEA=.027.

^d^Eigenvalue 2.777,

^e^Eigenvalue 1.466.

^f^Eigenvalue .927.

^g^Eigenvalue .715.

^h^Eigenvalue .668.

^i^Eigenvalue .640.

^j^Eigenvalue .437.

^k^Eigenvalue .369.

*P<.05*.*

EFA yielded a 2-factor solution with adequate model fit. The RMSEA and TLI indicated adequate model fit and CFI indicated good model fit (Table 4). The Kaiser criterion was met for the first 2 factors; eigenvalues of the third through eighth factor were <1. The first factor of the 2-factor solution consisted of the PTSD items and the second factor consisted of the functioning and resilience items. No cross-loadings were observed in this model.

Next, we conducted CFA to further compare the 2- and 3-factor model that resulted from EFA. Table 5 presents the model fit indices based on CFA of both aforementioned models. The model fit indices were similar for both models; the CFI and TLI indicated good model fit, the RMSEA acceptable model fit. As indicated by the significant χ^2^ difference test, the 2-factor model has worse model fit compared with the 3-factor model (χ^2^_2,n=1112_=13.63, *P*=.001). However, the difference in CFI is <0.01, indicating the 2-factor model does not have worse model fit. We selected the 2-factor model as the best-fitting model to our data, given the χ^2^ difference test is sensitive to sample size, the CFI difference is <.001, and it is more parsimonious and better interpretable at a conceptual level compared with the 3-factor model. The 2-factor model represents a clear distinction between negatively formulated outcomes (PTSD complaints) and positively formulated outcomes (psychosocial functioning and resilience) of PTEs. The positively formulated outcomes combine psychosocial functioning, social support, self-reliance and problem solving. We therefore propose to rename this factor psychosocial resources.

**Table 5:** Confirmatory factor analysis model fit indices (n=1112).

Model	χ^2^	*P* value	df^a^	CFI^b^	TLI^c^	RMSEA^d^
Two-factor solution	107.78	<.001	19	0.965	0.948	0.065
Three-factor solution	95.868	<.001	17	0.969	0.949	0.064

^a^df: degree of freedom.

^b^CFI: comparative fit index.

^c^TLI: Tucker-Lewis index.

^d^RMSEA: root mean square error of approximation.

### Internal Consistency

Interitem correlations of MIRROR’s PTSD complaints scale ranged between .28 and .48 with a mean of .34. All of the interitem correlations of the PTSD scale were in the recommended range of moderate magnitude of .15 to .50, indicating that this scale has high internal consistency in combination with a differentiated item set. Corrected item-total correlations for this scale ranged between .39 and .54 with a mean of .46, indicating that high scores on the PTSD items are associated with high scores on the overall PTSD scale of MIRROR. Cronbach alpha coefficient for MIRROR’s PTSD scale was .68.

Interitem correlations of MIRROR’s resilience scale ranged between .24 and .55, with a mean of .36. In addition, 1 out of 3 interitem correlations was higher than the recommended range of moderate magnitude of .15 to .50 (between “are you confident in yourself” and “are you able to deal with any problems you encounter”), indicating that this scale has high internal consistency in combination with a differentiated item set. Corrected item-total correlations ranged between .30 and .52 with a mean of .44, indicating that high scores on the resilience items are associated with high scores on the overall resilience scale of MIRROR. Cronbach alpha coefficient for MIRROR’s resilience scale was .62.

### Convergent and Divergent Validity

Pearson correlations between MIRROR and reference measures are presented in Table 6. MIRROR’s PTSD scale showed strongest correlations with PTSD as measured with the PCL-5, followed by a lower but still substantial correlation with psychological complaints as assessed with the DASS-21. The weakest correlations were observed between PTSD symptom severity as assessed with MIRROR and psychological resilience and positive mental health. MIRROR’s resilience scale showed strongest correlation with psychological resilience (RES), followed by a slightly lower correlation with positive mental health, psychological complaints (DASS-21), and PTSD (PCL-5). MIRROR’s functioning item showed strongest correlations with psychological complaints (DASS-21) followed by PTSD (PCL-5) with lower correlations with positive mental health (MHC-SF) and psychological resilience (RES). In conclusion, the correlational structure indicates good convergent and divergent validity of MIRROR’s PTSD subscale. The correlational structure with regard to MIRROR’s resilience scale and functioning item indicates adequate convergent and divergent validity.

**Table 6:** Correlations between Mobile Insight in Risk, Resilience and Online Referral (MIRROR) subscales and reference measures (n=663).

MIRROR	PTSD^a^	*P* value	Resilience	*P* value	Functioning	*P* value
PCL-5^b^	.665	<.001	–.507	<.001	–.442	<.001
DASS-21^c^	.486	<.001	–.539	<.001	–.449	<.001
RES^d^	–.265	<.001	.612	<.001	.279	<.001
MHC-SF^e^	–.259	<.001	.603	<.001	.319	<.001

^a^PTSD: posttraumatic stress disorder.

^b^PCL-5: PTSD Checklist for DSM-5.

^c^DASS-21: Depression Anxiety Stress scale.

^d^RES: Resilience Evaluation Scale.

^e^MHC-SF: Mental Health Continuum Short Form.

### Classification Quality

We expected respondents with the red MIRROR outcome to report more PTSD symptoms and depression, anxiety, and stress complaints; lower psychological resilience; and positive mental health compared with respondents with the green and orange MIRROR outcome. Table 7 presents the means and standard deviations on the reference measures for each MIRROR outcome category. Figure 1 shows the classification percentages on reference measures for each MIRROR outcome category. Both Table 7 and Figure 1 show that respondents with the red MIRROR outcome category report higher complaints and lower psychological resilience and positive mental health compared with the orange and green MIRROR outcome category.

**Table 7:** Means and standard deviations of reference measures for each Mobile Insight in Risk, Resilience and Online Referral (MIRROR) outcome category (n=663).

MIRROR^a^ outcome category (n)	Green (n=24), mean (SD)	Orange (n=200), mean (SD)	Red (n=439), mean (SD)
PTSD^b^ (PCL-5^c^)	18.04 (12.49)	36.09 (15.77)	46.13 (14.04)
Depression (DASS-21^d^)	4.08 (8.10)	11.73 (11.54)	19.66 (11.54)
Anxiety (DASS-21)	5.25 (6.72)	14.03 (10.27)	18.04 (10.30)
Stress (DASS-21)	10.42 (7.32)	17.60 (9.20)	22.49 (9.37)
Psychological resilience (RES^e^)	25.58 (5.11)	22.04 (6.02)	18.82 (7.15)
Positive mental health (MHC-SF^f^)	50.0 (12.05)	43.11 (14.89)	31.42 (14.28)

^a^MIRROR: Mobile Insight in Risk, Resilience and Online Referral.

^b^PTSD: posttraumatic stress disorder.

^c^PCL-5: PTSD Checklist for DSM-5.

^d^DASS-21: Depression Anxiety Stress Scale.

^e^RES: Resilience Evaluation Scale.

^f^MHC-SF: Mental Health Continuum Short Form.

We conducted several 1-way between-groups ANOVAs to investigate the difference in mean scores on the reference measures between MIRROR outcome categories. As can be seen, negative outcomes were highest for the red MIRROR outcome category and positive outcomes highest for the green outcome category. The ANOVA results are shown in Table 8. We found significant differences in PTSD symptoms; depression anxiety, and stress; psychological resilience; and positive mental health between groups. Post hoc tests revealed that PTSD symptoms and depression, anxiety, and stress complaints were significantly different between all groups (*P*<.001). Psychological resilience was significantly higher for the green and orange MIRROR outcome category compared with the red category (*P*<.001). It was also significantly higher for the green category compared with the orange category (*P*=.01). Positive mental health was significantly higher for the green and orange category compared with the red category (*P*<.001). There was no significant difference between the green and orange category (*P*=.07).

**Table 8:** One-way between-groups analyses of variance with Mobile Insight in Risk, Resilience and Online Referral (MIRROR) outcome categories and reference measures.

Analysis of variance	*F*-test	Cohen *d*	df^a^ between groups	df within groups	*P* value
PTSD^b^ symptoms^c^	73.32	.168	2	62.90	<.001
Depression^c^	65.21	.136	2	65.81	<.001
Anxiety^c^	42.48	.072	2	67.37	<.001
Stress	34.15	.094	2	660.0	<.001
Psychological resilience^c^	30.13	.068	2	65.44	<.001
Positive mental health	57.79	.069	2	660.00	<.001

^a^df: degree of freedom.

^b^PTSD: posttraumatic stress disorder.

^c^The assumption of equal variances was violated. Therefore, the Welch *F*-test and Games-Howell post hoc test were used.

## Discussion

### Principal Findings and Comparison With Prior Work

The purpose of this study was to evaluate the use and psychometric and classification properties of MIRROR. MIRROR is an innovative web-based self-help test to identify individuals who develop psychological complaints after a PTE, encourage them to seek help, and support self-reliance. Our results indicated that MIRROR is a valid and reliable self-help test to identify negative outcomes (PTSD core symptoms) and positive outcomes (psychosocial functioning and resilience). MIRROR is able to correctly classify respondents according to their PTSD complaints and scores on reference measures. During the study period, 87.95% (2247/2555) of respondents who started MIRROR completed it.

We found that MIRROR’s presupposed model of 3 factors (PTSD symptoms, psychosocial functioning, and resilience) did not fit our data best. Instead, a 2-factor solution showed good model fit, conceptual meaning, and maximum parsimony. This model separates MIRROR’s PTSD items from the functioning and resilience items (social support, self-reliance, and problem solving). In retrospect, the grouping of the functioning and resilience items is not entirely surprising. If we assume stress to be the result of an imbalance between perceived external and internal demands and perceived personal and social resources [62], it is likely that this distinction between demands and resources is reflected in the way people cope with PTEs. We propose to call the factor psychosocial resources. In accordance with this distinction, the 2-factor model clearly separates negative (PTSD complaints) and positive (psychosocial resources) outcomes of PTEs. This is in line with the general notion that PTSD and psychosocial resources are separate constructs [63-65].

The convergent and divergent validity of MIRROR is supported by the correlations that were found between MIRROR and the reference measures. The results indicate good convergent and divergent validity for MIRROR’s PTSD items. As expected, MIRROR’s PTSD showed strongest correlations with PTSD (assessed with the PCL-5), followed by a lower but substantial correlation with psychological complaints (measured with the DASS-21). MIRROR’s PTSD items showed low correlations with positive reference measures (assessed with the RES and MHC-SF). The results indicate adequate convergent and divergent validity for MIRROR’s resilience items but less distinct than MIRROR’s PTSD. MIRROR’s resilience items showed strongest correlations with psychological resilience, followed by slightly lower but substantial correlations with the other reference measures. The results in this study correspond with the finding of Van der Meer et al [43] who found the RES total scale to be positively associated with established measures for resilience, self-esteem, self-efficacy, and global functioning and negatively associated with PTSD symptoms. Furthermore, the different patterns of correlations for MIRROR’s PTSD and resilience scales agrees with the notion that PTSD and resilience are two separate constructs [63-65]. MIRROR’s functioning item showed the strongest correlation with psychological complaints and PTSD and lower correlations with the positive reference measures. This indicates adequate convergent and divergent validity. The factor analyses revealed that functioning belongs to the resilience items of MIRROR. However, the correlation between MIRROR’s functioning item and psychological complaints and PTSD is in line with studies that show that psychosocial functioning can be impaired by psychological complaints [64,66,67].

We found that both MIRROR’s PTSD and resilience scales show good internal consistency. The Cronbach alpha coefficients for these scales are relatively low (.68 and .62, respectively), but this is not unusual given the (intentionally) short scales of MIRROR and given that Cronbach alpha is a function of scale length [61]. Because MIRROR contains only few items, we calculated interitem and item-total correlations. The results indicate that both scales have high internal consistency and that high scores on the items are associated with high scores on the overall scales.

MIRROR was able to correctly classify respondents into green (no further action needed), orange (encourage self-monitoring), or red (encourage seeking consultation) outcome categories and advice compared with the other measures. Results showed that respondents with a red outcome reported having more severe PTSD symptoms; more severe depression, anxiety, and stress complaints; and lower psychological resilience and positive mental health compared with respondents with a green or orange outcome. The occurrence of PTSD and other stress-related complaints like depression following traumatic exposure is in line with former results [68]. It is important to recognize that MIRROR is specifically evaluating the risk of developing PTSD instead of other mental health outcomes of PTEs such as depression, anxiety, and substance abuse. If a respondent experiences low functioning, they will receive advice to seek consultation with their general practitioner despite the level of their PTSD complaints. This is based on the assumption that low functioning but no PTSD complaints may indicate that other problems could be at hand such as depression, anxiety, or substance abuse. Importantly, MIRROR appears to adequately identify users with more severe complaints and validly advises them to seek help. Our results seem to underline the relevance of including the factor “time since the event” in MIRROR’s algorithm. According to the PCL-5, 54.5% (109/200) of the respondents with the orange outcome had a provisional PTSD diagnosis. However, their complaints could still diminish, considering the event happened only recently for these respondents and research has shown that in most individuals complaints usually diminish over time [1,2,11]. Therefore, in accordance with international guidelines [14], respondents with the orange outcome are advised to monitor how their complaints develop (by setting a reminder to use MIRROR again in 2 weeks).

The evaluation of MIRROR’s use with Google Analytics showed that the number of users of MIRROR was substantial (n=2555), and the completion rate was high (2247/2555, 87.95%). These results are in line with former studies on apps assessing and monitoring mental health after PTEs indicating high use [29,30,36] and high completion rate [49]. In general, the follow-up options were visited less frequently (161 to 411 unique visits) than the outcome and advice page (2247 unique visits). A reason for this could be that receiving MIRROR’s outcome and advice is sufficient initial support for people who have experienced a PTE, providing insight into how they are coping. A total of 28.7% (113/194) of respondents who were advised to complete MIRROR again in 2 weeks immediately set a reminder, suggesting MIRROR is able to support self-monitoring. Unfortunately, this study’s design and considerations of ethical nature did not enable us to assess use in more depth.

### Future Research and Limitations

Although guidelines on screening for PTSD complaints and postdisaster psychosocial care are widely available [7,15,69-71], the challenge remains how to reach and identify people at risk of developing psychological complaints after a PTE on a large scale. Future research could focus on investigating the implementation of MIRROR on a larger scale—for example, after terrorist attacks or natural disasters. Literature is inconclusive about the benefits versus disadvantages of formal screening of an entire population after a disaster or crisis [14,15,69,72]. Because of limited evidence of effectivity and sensitivity of screening, organizational efforts related to screening, and the often scarce resources available [25,73], it is generally not recommended to perform formal screening of complaints among all involved people following incidents. At the same time, we know that early recognition and timely referral to help are essential for preventing and treating traumatic stress symptoms. This is supported by evidence of the effectiveness of early psychological interventions for individuals prescreened with traumatic stress symptoms shortly following trauma and no benefits in those not prescreened for these symptoms [16]. Mobile apps such as MIRROR can make a contribution to solving the screening dilemma by supporting low key, accessible, and easy-to-use self-assessment and -monitoring. In this view, MIRROR could be implemented as a first step in the support for people who have experienced a PTE, before having to consult professional care [29,36]. MIRROR might lower the barrier to seek help given its open accessibility and anonymity. Future research could focus on acquiring longitudinal data of MIRROR to assess the development of complaints, functioning, and resilience over time and establish MIRROR’s ability to correctly classify users accordingly. Also, qualitative research might clarify what actions users take as a result of MIRROR’s personal advice.

Our study has some limitations. In our validation sample, 74.2% (492/663) of respondents were female, and 45.3% (300/663) of respondents were aged between 21 and 40 years. This could lead to selection bias and limited generalizability of the results, which is common with open internet surveys [74]. However, our sample is a specifically targeted sample because it consisted of visitors of the website of Victim Support Netherlands. Considering website visitors were automatically led to MIRROR when searching for information regarding stress reactions following a PTE, a high prevalence of psychological complaints after traumatic exposure in our sample could be expected. Moreover, research has shown that women have a higher risk of developing PTSD compared with men [75], they are more likely to seek medical or health-related information online [76], and young people use the internet as their main source of information, and this is also true for mental health concerns [77,78]. This demonstrates that the targeted sample was reached. The main strength of this study is by comparing MIRROR to more broadly used reference measures, it contributes to the highly needed evidence base of mobile apps with the potential to improve timely identification of psychological complaints [29,30,79].

### Conclusions

This study shows that MIRROR is a psychometrically sound, anonymous, and easily accessible self-help test for people who have experienced a PTE. It is able to identify both negative (PTSD symptoms) and positive (psychosocial resources) outcomes of PTEs and classify respondents in accordance with reference measures. This study will hopefully contribute to enhancing adequate and timely identification of people who suffer from psychological complaints after PTEs.

## Appendix

Multimedia Appendix 1 Overview of outcomes in the Mobile Insight in Risk, Resilience, and Online Referral (MIRROR) instrument.

Multimedia Appendix 2 Sample characteristics on reference measures and demography (n=663).

## Abbreviations

ANOVA: analysis of variance
CFA: confirmatory analysis
CFI: comparative fit index
DASS-21: Depression Anxiety Stress scale
DSM-5: Diagnostic and Statistical Manual of Mental Disorders, Fifth Edition
EFA: exploratory factor analysis
eMEN: e-Mental health innovation and transnational implementation platform North West Europe
GAF: Global Assessment of Functioning
ICD-10: International Statistical Classification of Diseases and Related Health Problems, Tenth Revision
LEC-5: Life Events Checklist for DSM-5
MHC-SF: Mental Health Continuum Short Form
MIRROR: Mobile Insight in Risk, Resilience and Online Referral
NICE: National Institute for Health and Care Excellence
PCL-5: PTSD Checklist for DSM-5
PTE: potentially traumatic event
PTSD: posttraumatic stress disorder
RES: Resilience Evaluation Scale
RMSEA: root mean square error of approximation
TENTS: The European Network for Traumatic Stress
TLI: Tucker-Lewis index
WLSMV: means and variance adjusted weighted least square
