# Correction: Mobile Insight in Risk, Resilience, and Online Referral (MIRROR): Psychometric Evaluation of an Online Self-Help Test

**DOI:** 10.2196/29776

**Published:** 2021-06-04

**Authors:** Merel Marjolein van Herpen, Manon A Boeschoten, Hans te Brake, Niels van der Aa, Miranda Olff

**Affiliations:** 1 ARQ Centre of Expertise for the Impact of Disasters and Crises Diemen Netherlands; 2 Department of Psychiatry Amsterdam Neuroscience & Public Health Amsterdam University Medical Center Amsterdam Netherlands; 3 ARQ Centre '45 Oegstgeest/Diemen Netherlands; 4 ARQ National Psychotrauma Centre Diemen Netherlands

In “Mobile Insight in Risk, Resilience, and Online Referral (MIRROR): Psychometric Evaluation of an Online Self-Help Test” (J Med Internet Res 2020;22(9):e19716) one correction was made. This does not affect the analysis nor does it affect the interpretation or presentation of the results in the study.

In the “Methods” section, under “Measures” and “Depression, Anxiety and Stress”, the following phrase appears:

A 5-point response scale measures the extent to which each state has been experienced over the past week ranging from 0 (not at all) to 4 (most certainly).

This has been replaced by the following:

A 4-point response scale measures the extent to which each state has been experienced over the past week ranging from 0 (did not apply to me at all) to 3 (applied to me very much, or most of the time).

The correction will appear in the online version of the paper on the JMIR Publications website on June 4, 2021, together with the publication of this correction notice. Because this was made after submission to PubMed, PubMed Central, and other full-text repositories, the corrected article has also been resubmitted to those repositories.

