# Supplementary material for: Mobile Insight in Risk, Resilience, and Online Referral (MIRROR): Psychometric Evaluation of an Online Self-Help Test
Source: J Med Internet Res. 2020 Sep 25;22(9):e19716. doi: 10.2196/19716 (PMC7547397; doi:10.2196/19716)
Supplement: Multimedia Appendix 1 [file jmir_v22i9e19716_app1.docx]

## Multimedia appendix 1

### Overview of MIRROR’s outcomes.

| **Time since trauma: less than one week** | | | |
| --- | --- | --- | --- |
|  | PTSD low | PTSD moderate | PTSD high |
| Functioning high | Green | Orange | Orange |
| Functioning moderate | Orange | Orange | Orange |
| Functioning low | Red | Red | Red |
| **Time since trauma: between one and four weeks** | | | |
|  | PTSD low | PTSD moderate | PTSD high |
| Functioning high | Green | Green | Orange |
| Functioning moderate | Green | Orange | Red |
| Functioning low | Red | Red | Red |
| **Time since trauma: more than four weeks / repetitively** | | | |
|  | PTSD low | PTSD moderate | PTSD high |
| Functioning high | Green | Green | Red |
| Functioning moderate | Green | Red | Red |
| Functioning low | Red | Red | Red |

*Notes. PTSD complaints: 4 items, functioning: 1 item and resilience: 3 items. In total, MIRROR provides 27 unique types of advice texts, which take all levels of PTSD complaints, functioning and resilience and time passed since the event into account.*
