# Supplementary material for: Mobile Insight in Risk, Resilience, and Online Referral (MIRROR): Psychometric Evaluation of an Online Self-Help Test
Source: J Med Internet Res. 2020 Sep 25;22(9):e19716. doi: 10.2196/19716 (PMC7547397; doi:10.2196/19716)
Supplement: Multimedia Appendix 2 [file jmir_v22i9e19716_app2.docx]

## Multimedia appendix 2

### Sample characteristics on reference measures and demography (n = 663).

|  |  | | **M** | **SD** | **Range** |
| --- | --- | --- | --- | --- | --- |
| **PTSD (PCL-5)** |  | |  |  |  |
| Total score |  | | 42.09 | 15.91 | 5 – 77 |
| Intrusion |  | | 11.02 | 4.59 | 0 – 20 |
| Avoidance |  | | 4.22 | 2.26 | 0 – 8 |
| Alterations in cognition and mood |  | | 13.98 | 6.82 | 0 – 28 |
| Negative alterations in arousal and reactivity |  | | 12.87 | 5.08 | 0 – 24 |
| **Depression, anxiety and stress (DASS-21)** |  | |  |  |  |
| Depression |  | | 16.70 | 11.84 | 0 – 42 |
| Anxiety |  | | 16.37 | 10.56 | 0 – 42 |
| Stress |  | | 20.58 | 9.71 | 0 – 42 |
| **Psychological resilience (RES)** |  | |  |  |  |
| Self-efficacy |  | | 13.42 | 4.75 | 0 – 24 |
| Self-confidence |  | | 6.62 | 2.99 | 0 – 12 |
| **Positive mental health (MHC-14)** |  | |  |  |  |
| Emotional wellbeing |  | | 8.26 | 3.71 | 0 – 15 |
| Social wellbeing |  | | 11.08 | 6.15 | 0 – 25 |
| Psychological wellbeing |  | | 16.27 | 7.36 | 0 – 30 |
| **Gender** | **N (%)** |  | |  |  |
| Female | 492 (74.2) |  | |  |  |
| Male | 170 (25.6) |  | |  |  |
| Prefer not to answer | 1 (0.2) |  | |  |  |
| **Age** | **N (%)** |  | |  |  |
| 14 – 20 years | 72 (10.9) |  | |  |  |
| 21 – 30 years | 182 (27.5) |  | |  |  |
| 31 – 40 years | 118 (17.8) |  | |  |  |
| 41 – 50 years | 124 (18.7) |  | |  |  |
| 51 – 60 years | 106 (16.0) |  | |  |  |
| 61 – 70 years | 51 (7.7) |  | |  |  |
| 71+ years | 10 (1.5) |  | |  |  |
| **Education** | **N (%)** |  | |  |  |
| Primary | 19 (2.9) |  | |  |  |
| Pre-vocational secondary | 97 (14.6) |  | |  |  |
| Secondary or vocational | 234 (35.3) |  | |  |  |
| Higher | 299 (45.1) |  | |  |  |
| Prefer not to answer | 14 (2.1) |  | |  |  |
| **Marital status** | **N (%)** |  | |  |  |
| Single | 284 (42.8) |  | |  |  |
| Married/cohabiting with children | 170 (25.6) |  | |  |  |
| Married/cohabiting without children | 115 (17.3) |  | |  |  |
| Steady relationship | 60 (9.0) |  | |  |  |
| Prefer not to answer | 34 (5.1) |  | |  |  |
